# Brain-Wave Music: A Personalized Approach to Improving Anxiety Resistance

**DOI:** 10.1155/np/3500022

**Published:** 2025-11-26

**Authors:** Haohan Yang, Yan Li, Haoyu Bian, Chenxi Qiu, Xinjian Su, Jiaxian Chen, Roberto Rodriguez-Labrada, Jing Lu, Dezhong Yao

**Affiliations:** ^1^Yingcai Honors College, University of Electronic Science and Technology of China, Chengdu 611731, Sichuan, China; ^2^Key Lab for NeuroInformation of Ministry of Education, School of Life Science and Technology, University of Electronic Science and Technology of China, Chengdu 611731, Sichuan, China; ^3^Cuban Neuroscience Center, La Habana 11600, Cuba; ^4^China-Cuba Belt and Road Joint Laboratory on Neurotechnology and Brain-Apparatus Communication, University of Electronic Science and Technology of China, Chengdu 611731, Sichuan, China

**Keywords:** anxiety, EEG, music

## Abstract

**Introduction:**

Music has long been recognized for its potential to modulate anxiety resistance at the population level. However, there is a lack of an auditory method that performs general and effective in enhancing anxiety resistance. Here we investigate the impact of a specific music that directly reflects brain activity (brain-wave music [BWM]) on anxiety resistance and its underlying neural mechanisms.

**Method:**

A two-phase experimental protocol was designed utilizing the ToS anxiety induction paradigm to examine the efficacy of BWM in enhancing anxiety resistance. In Phase 1, resting-state EEG recordings were initially collected from 70 participants, followed by a standardized anxiety induction procedure involving auditory cues, to validate the effectiveness of the anxiety induction paradigm and establish baseline anxiety resistance for each participant. Phase 2, designed based on Phase 1 and conducted 24 h later, further investigated the neural mechanisms underlying anxiety regulation through brain-music intervention. Participants were randomly allocated into three groups: BWM (*n* = 30) group exposed to personalized EEG generated music, Preferred Music Control (PMC, *n* = 20) group to self-selected music, and silent control (SC, *n* = 20) to no auditory input. The anxiety induction procedure was then repeated in all groups. Anxiety levels were assessed through the state anxiety inventory (SAI) in both phases.

**Results:**

Compared to Phase 1 resting-state baseline, the BWM group exhibited significantly reduced SAI scores in Phase 2, accompanied by enhanced prefrontal theta oscillations and functional connectivity between the prefrontal cortex, parietal lobe, and auditory cortex. No significant changes were observed in the other groups.

**Discussion:**

These findings suggest that BWM effectively promotes anxiety resistance by facilitating network connectivity between the prefrontal and multisensory regions. Moreover, this study highlights BWM as a novel and promising method for emotional regulation.

## 1. Introduction

Anxiety, as a negative emotional state, is prevalent across various age groups. Previous studies have shown that prolonged anxiety can lead to a range of health issues, such as insomnia, cardiovascular diseases, and limitations in social functioning [1–3]. In recent years, an increasing amount of research has focused on anxiety prevention, specifically on enhancing individuals' anxiety resistance [4–6].

Music has been widely recognized and utilized in medical settings as an effective nonpharmacological intervention for regulating anxiety, offering a therapeutic approach with minimal side effects and broad applicability [7, 8]. Previous studies have demonstrated that listening to music has positive effects across various settings, including physiological arousal (e.g., heart rate, blood pressure, and hormone levels) and psychological stress experiences (e.g., restlessness, anxiety, and nervousness) [9].

In parallel, related studies have also indicated that music has a regulatory effect in improving resistance to different subtypes of anxiety, including but not limited to anxiety in patients undergoing medical treatment and social anxiety [10–14]. For instance, Ergin et al. and Liu et al. reported that music listening significantly alleviated anxiety, respectively [15, 16]. Chlan et al. [17] highlighted that patient-directed music was effective in reducing anxiety, underscoring the critical role of individual preference and personalization. However, Kakar et al. [18] found no significant reduction in anxiety following nonpersonalized music interventions in the similar patients. Simultaneously, studies by Cheung et al. [19] and Burrai et al. [20] also reported no significant differences in anxiety between intervention and control groups at follow-up, further indicating that the inconsistency in outcomes may stem from a lack of alignment between the music used and individual listeners' preferences or needs [19–21]. Given these inconsistencies, it is essential to identify an effective, stable, and personalized auditory approach that can reliably regulate anxiety resistance across individuals and contexts.

Among various music-based interventions, brain-wave music (BWM) has garnered increasing attention in recent years due to its unique ability to directly reflect and resonate with neural activity patterns and individuation. Prior research has demonstrated that BWM not only enhances cognitive functions such as attention, memory, and executive control but also plays a significant role in improving sleep quality and overall well-being [22–24]. These findings underscore the versatility of BWM as a therapeutic intervention, offering a promising non-pharmacological approach to mental health management. Its ability to modulate neural activity and promote a state of relaxation and balance suggests that it could serve as a personalized treatment for a wide range of psychological and physiological conditions.

Building upon these prior findings, the present study seeks to further investigate the impact of BWM on human resistance to anxiety, with a particular focus on the underlying neural mechanisms. Previous research has established that theta oscillations in the prefrontal cortex play a critical role in modulating both anxiety and depressive states [25–27]. By exposing participants to their personalized BWM prior to the anxiety-induction phase, we aimed to evaluate its impact on emotional regulation and the neural mechanisms underlying anxiety-related responses.

## 2. Materials and Methods

### 2.1. Participants

This study was conducted in accordance with the ethical guidelines of the Declaration of Helsinki and was approved by the Ethics Committee of the University of Electronic Technology and Science of China (Ethics approval code: No. 106142023121227791). Prior to participation, all individuals provided written informed consent, acknowledging their voluntary participation and understanding of the study procedures.

A total of 70 participants were recruited for this study and subsequently assigned to one of three experimental groups. The BWM group (*n* = 30, 17 males, age: 22.17 ± 2.18) consisted of individuals exposed to personalized BWM. The preferred music control (PMC, *n* = 20, 12 males, age: 22.33 ± 3.93), included participants who listened to self-selected music. The silent control group (SC, *n* = 20, 15 males, age: 22.8 ± 2.36), comprised individuals who did not receive any musical intervention. All participants were right-handed as assessed by the Edinburgh Handedness Inventory [28].

All participants completed standardized psychometric assessments, including the Basic Information Questionnaire, Self-Rating Anxiety Scale (SAS) [29], and State–Trait Anxiety Inventory (STAI) [30], at the same stage of the experiment to assess baseline anxiety levels, demographic characteristics, and ensure intergroup homogeneity. Particularly, the State Anxiety Inventory (SAI), component of the STAI, was administered to measure participants' anxiety levels across different experimental phases.

Individuals were excluded for: (1) history of neurological disorders, psychiatric conditions, or substance abuse; (2) any psychotropic medications use within the past 3 months.

### 2.2. Experimental Protocol

**Figure 1:**
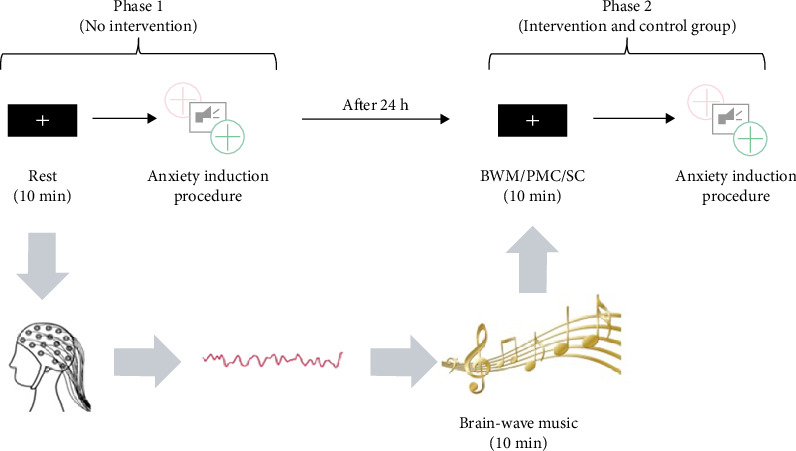
The figure illustrates the experimental procedure. In Phase 2, the brain-wave music for BWM group was generated by transforming the data recorded from Fp1 and Fp2 electrodes during the rest part of Phase 1.

The protocol of the experiment is shown in Figure 1. In Phase 1, the experiment began with a 10-min resting-state EEG recording, which was used to establish a baseline for the participants' neural activity. During this period, participants were instructed to focus on a white cross displayed in the center of the screen, with no music playing through their headphones.

The participants then underwent an auditory anxiety induction task, designed to elicit anxiety responses in line with the principles of the *ToS* (*Threat of Scream*) anxiety induction theory [31]. We utilized seven different scream recordings, sourced from women and children. These scream sounds were selected based on their capacity to evoke strong emotional responses. The task involved 84 instances of anxiety induction, all stimuli were presented using E-Prime 3.0 (Psychology Software Tools, Pittsburgh, PA, USA, https://pstnet.com/products/e-prime/, accessed on 31 July 2021).

The anxiety induction procedure began with the presentation of a colored cross in the center of the screen. The cross appeared in two different colors: red and green. The red cross signaled to participants that they would likely hear a scream after a 3-s delay, whereas the green cross indicated that no sound would follow. The red cross was presented in 50% of the trials. Among these “red cross” trials, the red cross appeared without an accompanying scream in 10% of the cases. At the end of the anxiety induction phase, participants were again asked to complete a second anxiety scale, the SAI scale.

24 h after Phase 1, the experiment proceeded to Phase 2, the participants were divided into the following three groups:

BWM Group: Participants in this group were exposed to BWM, an auditory stimulus specifically designed to interact with individual neural activity. The music was generated using real-time EEG data, specifically from the Fp1 and Fp2 channels recorded during the resting-state session in Phase 1. By analyzing each participant's baseline neural patterns, the music was tailored to resonate with their unique brain-wave profiles [23], where EEG amplitude, period, and power recorded from the Fp1 and Fp2 channels were respectively mapped to musical pitch, rhythm, and intensity following the framework proposed by Wu et al. [24] and Lu et al. [23]. Importantly, participants were not informed that the auditory stimulus was derived from their own EEG data. All groups received identical standardized instructions, which only described the session as an auditory experience designed for relaxation. This procedure was adopted to minimize potential expectancy or placebo effects and to ensure that any observed differences in anxiety regulation were attributable to the intrinsic properties of the stimuli rather than to participants' beliefs or awareness. The aim of this approach was to enhance synchronization between natural brain oscillations and the auditory input.

PMC: Participants in this group listened to a preferred music genre of their own choice, selected prior to the experiment from options including classical, pop, or folk music. This group served as an active control to the BWM group.

SC: Participants in the SC group followed the same experimental protocol as in Phase 1, but without any additional auditory stimulation. This group served as a negative control to the BWM group.

Participants first underwent a 10-min music listening session according to their assigned group, followed by the same anxiety induction paradigm.

The SAI component of the STAI was recorded at multiple time points: before and after Phase 1, following the resting-state EEG recording in Phase 2, and after the anxiety-induction task in Phase 2.

### 2.3. EEG Recording and Data Preprocessing

EEG was recorded using an active system (ActiCap, Brain Products, Gilching, Germany). The EEG cap worn by participants had 63 Ag/AgCl electrodes placed according to the International 10–20 system, and data was sampled at 500 Hz and bandpass filtered from 0.1 to 40 Hz. We used “FCz” as reference electrode.

Offline processing of the EEG data was performed in MATLAB (R2016a; Mathworks, Natick, MA, USA) using the EEGLAB toolbox [32] (https://sccn.ucsd.edu/eeglab/index.php [accessed on 10 February 2022]) and Brainstorm software [33] (https://neuroimage.usc.edu/brainstorm/ [accessed on 10 February 2022]). Then, the data were transformed to zero reference using the EEGLAB toolbox [34]. Continuous EEG data were high-pass filtered above 1 Hz and low-pass filtered under 40 Hz. Subsequently, we extracted the rest-state EEG data. Then, the independent component analysis was applied to identify and remove eyeblinks and movements. After ocular correction, traces were scanned for artifacts and epochs with deflections exceeding ±60 µV marked and excluded.

### 2.4. Power Spectral Density (PSD) Analysis and Behavioral Analysis

We selected AF3 and AF4 electrodes, which have been shown in neuroimaging studies to be associated with emotional processing located in prefrontal lobe [35–38]. Subsequently, we focused on the theta band oscillations (4–8 Hz), as research has shown that this frequency range is associated with anxiety and depressive emotions [25–27].

We calculated the PSD of the overall 10-min resting-state recordings for each subject of both phases using Brainstorm, with a window length of 10 s and a 50% window overlap ratio. Subsequently, we performed a paired *t*-test on the PSD of different electrodes for Phase 1 and Phase 2 resting-state recordings.

Anxiety resistance levels were assessed using the difference in SAI scores measured before and after the anxiety induction procedure as shown in Figure 1, with lower values indicating greater anxiety resistance. A correlation analysis was performed between the resting-state PSD and the anxiety resistance measured in Phase 2 of BWM.

### 2.5. Functional Connectivity Analysis

To study the synchrony between different neurons, we used coherence (mscoh) in Brainstorm to extract coherence features between paired nodes. We also performed a group-level paired *t*-test on the coherence for Phase 1 and Phase 2 resting-state recordings and conducted a correlation analysis between the Phase 2 brain network connectivity features and the anxiety resistance improvement, which was calculated based on the reduction in the difference between SAI scores from Phase 1 to Phase 2.

## 3. Results

70 participants without mental or psychological diseases were finally included.

At baseline, the three groups did not differ significantly in age, sex distribution, SAS Score, TAI Score, or SCL-90 Score; all scale scores fell within normative ranges, indicating an absence of psychological disorders (Table 1).

### 3.1. Behavioral Results

**Figure 2:**
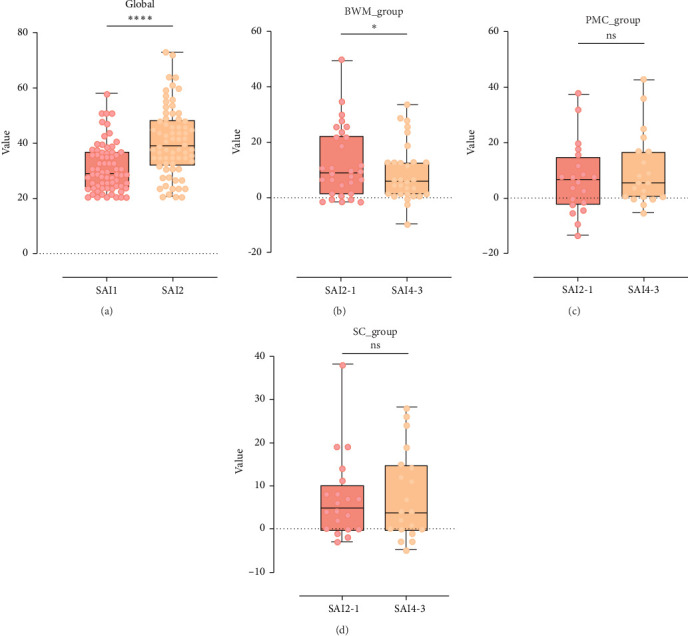
Comprehensive behavioral analysis across all participants and individual experimental groups. (a) Illustrates the SAI scores of the 70 participants before and after the anxiety-induction paradigm in Phase 1. (b) Shows that participants in BWM group exhibited a significant reduction in anxiety-induction intensity in Phase 2. In contrast, (c,d) indicate that no significant changes were observed in the PMC group or the SC group, respectively.

The behavioral results of the present study demonstrate that the auditory anxiety induction paradigm effectively elicits anxiety responses. As shown in Figure 2a, statistical analysis was performed to evaluate changes in participants' anxiety levels before and after the initial anxiety induction. Specifically, a Wilcoxon signed-rank test was applied to the SAI scores collected from 70 participants. The analysis revealed a statistically significant increase in anxiety levels following the auditory anxiety induction (*n* = 70, *Z* = 4.65, *p* < 0.001), indicating that the paradigm reliably induced anxiety across participants.

Furthermore, a comparative behavioral analysis was conducted across experimental groups to assess the impact of BWM on anxiety regulation. The results indicated that the increase in anxiety levels during the second anxiety induction session was significantly attenuated in the BWM group. As illustrated in Figure 2, participants in the BWM group demonstrated a statistically significant reduction, whereas no significant changes were observed in the PMC and SC groups (*n* = 30, *t* = 2.377, *p* < 0.05).

### 3.2. Analysis of Prefrontal Oscillation and Functional Connectivity

**Figure 3:**
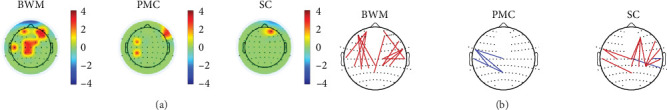
Analysis of power spectral density (PSD) and functional brain networks in theta band across groups. (a) Variation of PSD between the resting-state of Phase 1 and Phase 2. (b) Variation in functional connectivity, with red indicating an increase in connectivity and blue indicating a decrease. This figure highlights the increased theta oscillations in the prefrontal cortex and its connectivity with the frontal-parietal and frontotemporal lobe.

**Figure 4:**
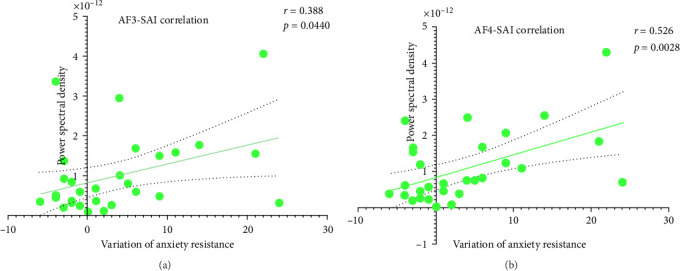
Correlation analysis between prefrontal power spectral density of BWM group in theta band and anxiety resistance improvement. (a and b) Shows the positive correlation between prefrontal PSD and variation of anxiety resistance.

As shown in Figure 3a, spectral analysis revealed a robust increase in theta-band PSD across the prefrontal cortex, temporal lobe, and parietal lobe following the BWM intervention. Correlation analyses were performed between these neurophysiological changes and behavioral outcomes within the BWM group. Prefrontal theta oscillation exhibited significant positive correlations with the improvement of anxiety resistance (AF3: *r* = 0.39, *p* < 0.05, AF4: *r* = 0.53, *p* < 0.01, Figure 4).

**Figure 5:**
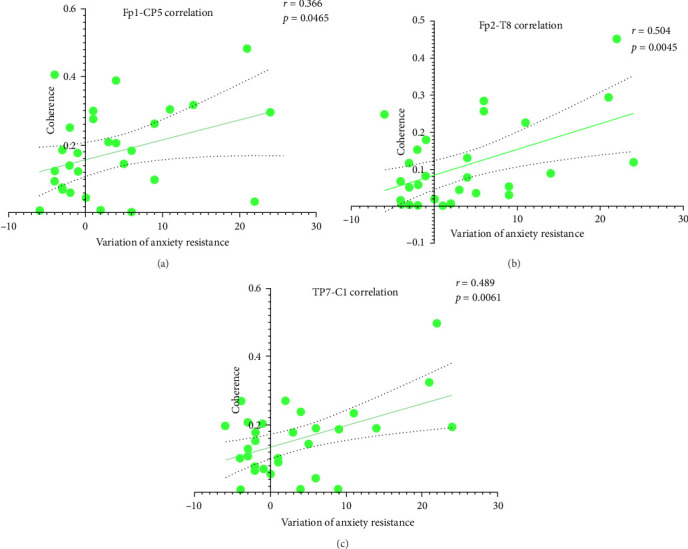
Correlation analysis between functional connectivity of BWM group in theta band and anxiety resistance improvement. Similar to Figure 3, positive correlations between connectivity strength and changes in anxiety resistance were observed across multiple pathways. (a) Shows the association between prefrontal–parietal connectivity strength and the variation in anxiety resistance. (b) Illustrates the relationship between prefrontal–auditory cortex connectivity and changes in anxiety resistance. (c) Depicts the association between auditory–parietal connectivity strength and the variation in anxiety resistance.

Functional connectivity analyses were conducted across three groups. These analyses revealed significant increases in theta-band coherence in the BWM group between the prefrontal cortex, parietal lobe, and auditory cortex (Figure 3b). Moreover, enhanced functional connectivity along the FP1–CP5 (*r* = 0.366, *p* < 0.05), FP2–T8 (*r* = 0.504, *p* < 0.01), and TP7–C1 (*r* = 0.489, *p* < 0.01) pathways demonstrated a significant correlation with improvement of anxiety resistance, as illustrated in Figure 5.

## 4. Discussion

Our study found that personalized BWM enhances individual resistance to anxiety. By comparing participants in the BWM group with those in the PMC and SC groups, the reduction in the difference in SAI scores supports the efficacy of BWM in significantly improving anxiety resistance. Simultaneously, the increase in theta-band PSD and coherence yielded critical insights into the neural mechanisms underlying this observed improvement. We further accessed the correlation between these neural adaptations and behavioral score.

Specifically, our results indicated a notable increase in brain activity within frontal, temporal, and parietal regions in the theta frequency band among participants in the BWM group compared to both control groups and baseline measurements taken during Phase 1 of our experimental design. This enhanced theta-band activity suggests heightened engagement and modulation of neural circuits associated with emotional regulation, cognitive processing, and attentional control, which are critical for managing anxiety responses effectively [25–27]. Notably, the enhancement in prefrontal theta power—localized primarily to regions associated with emotional regulation—supports the role of prefrontal oscillatory activity in attenuating anxiety-related neural responses [25].

Our findings demonstrate significant enhancements in functional connectivity between the prefrontal cortex, parietal lobe, and auditory cortex, revealing improved neural synchronization and communication within emotional regulation networks in BWM group. Meanwhile, we observed that connectivity patterns showing a distinct positive correlation with improved anxiety resistance. This neurophysiological evidence suggests that BWM facilitates integrative neural processes critical for maintaining emotional stability. The observed increase in theta-band connectivity between prefrontal and sensory regions is likely to reflect an integrative network synchronization mechanism. This enhanced synchronization suggests that BWM facilitates coherent communication across distributed neural systems, particularly between frontal regions involved in emotional regulation and sensory areas responsible for perceptual integration. Such large-scale network coordination may underlie the improved emotional stability and anxiety resistance observed in the BWM group.

In addition, meta-analytic evidence shows that conventional “one-size-fits-all” music interventions yield only moderate benefits and very high inter-study heterogeneity, indicating that the same playlist works for some listeners but not for others [39]. Personalized protocols address this mismatch by tailoring the soundtrack to each listener's psychophysiological profile; EEG-guided systems that classify moment-to-moment stress signatures and automatically recommend acoustically congruent pieces have already achieved larger reductions in anxiety and stress than generic selections [40, 41]. Our personalized BWM paradigm pushes personalization further: it simulates the individual's own brain oscillation, thereby maximizing phase- and frequency-specific alignment between stimulus and cortex. So, personalized BWM could facilities anxiety resistance levels by promoting theta oscillations and connectivity between frontal and multisensory regions. Together, these findings provide a neural mechanism for why personalized BWM offers a more potent route to boosting anxiety resistance than traditional, nonpersonalized music therapy.

Despite these outcomes, several limitations warrant careful consideration. First, while our results demonstrate that BWM has a distinct anxiolytic effect by modulating brain activity, the exact biochemical or molecular processes mediating this effect remain largely unexplored. Neurochemical pathways involving dopamine, serotonin, or GABA may underlie the observed oscillatory changes, this conjecture warrants direct empirical validation through pharmacological studies. Besides, future studies should aim to further elucidate the anxiolytic mechanisms of BWM—for instance, by incorporating real-time neurofeedback protocols to monitor and modulate neural activity in a closed-loop system and by conducting long-term follow-up assessments to gauge the durability of BWM-induced anxiety resistance.

## 5. Conclusions

This study provides evidence that BWM effectively mitigates anxiety responses during induced stress tasks, as demonstrated by both behavioral and neurophysiological indices. The observed reduction in anxiety shows the potential of this auditory intervention as a novel tool for enhancing anxiety resistance. Particularly, enhanced prefrontal theta-band oscillations and connectivity between the prefrontal and multisensory areas emerged as a key neural correlate of BWM-related anxiety resistance.

## Figures and Tables

**Table 1:** Demographic characteristics of three groups.

Characteristic	BWM_group	PMC_group	SC_group	*p*
Gender (male:female)	17:13	12:8	14:6	0.6299
Age (years ± s.d.)	22.17 ± 2.21	23.15 ± 4.03	22.80 ± 2.42	0.5368
SAS score	28.10 ± 5.03	29.55 ± 7.32	28.50 ± 6.19	0.8410
TAI score	33.87 ± 7.88	36.10 ± 9.37	34.30 ± 9.59	0.6900
SCL-90 score				
Somatization	13.73 ± 2.05	14.60 ± 3.10	13.30 ± 1.95	0.4191
Obsessive-compulsive	15.67 ± 3.81	15.95 ± 4.98	15.15 ± 4.09	0.8812
Interpersonal sensitivity	11.90 ± 2.71	12.40 ± 3.91	11.75 ± 3.09	0.8757
Depression	16.33 ± 3.87	18.10 ± 5.91	16.40 ± 4.66	0.5344
Anxiety	11.93 ± 1.84	12.60 ± 3.35	11.55 ± 2.39	0.3415
Hostility	6.80 ± 1.32	8.40 ± 3.15	7.15 ± 1.46	0.1154
Phobic anxiety	8.10 ± 1.81	8.60 ± 2.16	7.80 ± 1.44	0.4579
Paranoid ideation	7.03 ± 1.59	7.85 ± 2.39	7.20 ± 1.47	0.4496
Psychoticism	12.27 ± 2.33	13.90 ± 4.66	12.10 ± 2.65	0.6537
Other	9.17 ± 1.78	10.45 ± 2.91	8.75 ± 2.29	0.1085

## Data Availability

The data that support the findings of this study are available on request from the corresponding author. The data are not publicly available due to privacy or ethical restrictions.
